# Heart Rate Variability for the Prediction of Treatment Response in Major Depressive Disorder

**DOI:** 10.3389/fpsyt.2020.00607

**Published:** 2020-06-30

**Authors:** Kwan Woo Choi, Hong Jin Jeon

**Affiliations:** ^1^ Department of Psychiatry, Korea University College of Medicine, Seoul, South Korea; ^2^ Department of Psychiatry, Depression Center, Samsung Medical Center, Sungkyunkwan University School of Medicine, Seoul, South Korea

**Keywords:** heart rate variability, major depressive disorder, antidepressant treatment, treatment, diagnosis

## Abstract

Major depressive disorder (MDD) is one of the disabling diseases in the world-wide, and known to increase cardiac morbidity and mortality. Therefore, previous studies related heart rate variability (HRV) have been conducted to evaluate and diagnose MDD, and to predict treatment outcomes in patient with MDD. We reviewed extensively on the previous peer-reviewed publications associated with this issue, using Pub-Med. In this review article, we introduce the basic concept of HRV and HRV measures, and present several important findings associated with diagnosis and treatment prediction in MDD with using HRV parameters. Furthermore, we discuss the possible underlying mechanism of this phenomenon, and suggest several considerations for the future research.

## Introduction

Major depressive disorder (MDD) is one of the most disabling conditions, featured by depressive episodes lasting at least two weeks, over changes in mood, cognition, and vegetative symptoms (1). However, because MDD is a heterogenous condition, and patients with MDD exhibit multiple variable symptoms, which make the correct diagnosis difficult (2). Furthermore, although antidepressant medication has been considered as the first-line treatment for MDD, only 50% of patients are non-responsive to initial treatment, and it is difficult to predict future responsiveness of MDD at the time of beginning treatment (3). Therefore, it is necessary to develop a more reliable method to diagnose MDD and predict treatment responsivity in MDD patients.

Numerous research findings have proven that major depressive disorder (MDD) is strongly associated with elevated risk for the development and progression of cardiovascular diseases (4–13). Autonomic nervous system (ANS) dysfunction is considered one of the pathways linking MDD and negative CVD outcomes (14). Heart rate variability (HRV), levels of variability of the heart beat-to-beat interval over time, has been known to provide an index of ANS functioning including the sympathetic and parasympathetic system (15). In this brief review, we aim to describe a clinical overview of the HRV parameters, methodologic issues, and HRV research which found an association between HRV parameters and MDD diagnosis, and between baseline HRV parameters and MDD treatment responsivity.

## Materials and Methods

**Figure 1:**
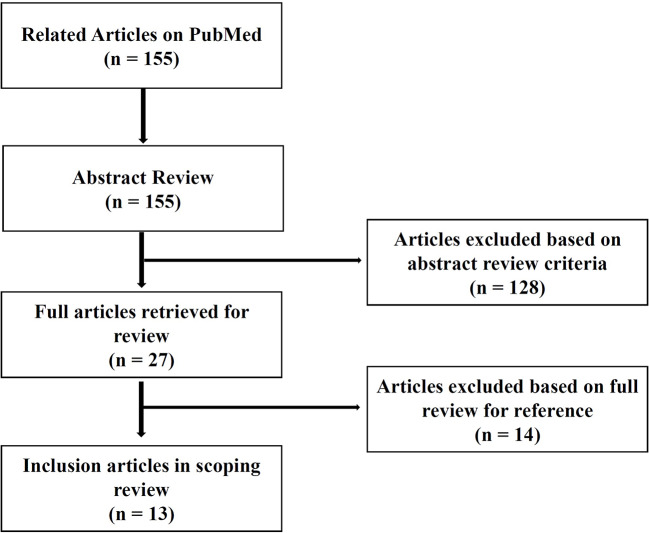
Flow chart of review selection process.

We performed a brief review of major publication on the diagnosis for MDD with using HRV use, and predictive value of HRV parameters for treatment response, especially in patients with MDD. A structured literature search was conducted from the PubMed data base until March 2020 (with no publication data limitations) (Arksey and O**’**Malley, 2005). Search terms and databases were determined in consultation with a health science librarian at Korea University and Samsung Medical Center. Relevant articles which were identified using the following keywords: **“**heart rate variability**”** and **“**major depressive disorder**”** and **“**diagnosis**”**; **“**heart rate variability**”** and **“**major depressive disorder**”** and **“**treatment**”**; **“**heart rate variability**”** and **“**major depressive disorder**”** and **“**treatment response**”**; **“**heart rate variability**”** and **“**depression**”** and **“**remission**”**. The retrieved title and abstracts were investigated for relevance for two reviewers (Kwan Woo Choi [KWC], and Hong Jin Jeon [HJJ]) using the following inclusion criteria: 1) the study focused on heart rate variability as the main outcome; 2) the study mainly focused on the diagnosis or treatment response of major depressive disorder (MDD); 3) the population of the study targeted adult people, who are older than 18 years old; 4) the article is written in English. In reviewing abstracts, citations were excluded from the review using the following criteria: 1) the study does not deal with specific HRV parameters; 2) the study was not written in English. The initial database search returned 155 database citations. 155 abstracts were selected to review for inclusion in the scoping review and 27citations qualified for full paper review. Following full paper review, seven articles were excluded as they did not meet inclusion criteria. The final set of 13 studies included (**Figure 1**).

## Heart Rate Variability Parameters

HRV is defined as fluctuation of the heart beat interval over time (16). Since the heart is dually innervated by the sympathetic and parasympathetic branches of the autonomous nervous system (ANS), subtle moment-to-moment changes in heart rate (HR) are qualitative indicators of ANS function (17). According to the reliable international guidelines, HRV parameters could be divided into two domains; frequency domain (spectral analysis) and time domain (non-spectral analysis) (**Table 1**) (15, 18).

**Table 1:** A brief description of the most relevant measures of heart rate variability.

Parameters	Description
***Time domain***	
SDNN	SD of the normal to normal intervals
RMSSD	Square root of the mean squared difference between successive RRs
NN50	The numbers of successive RR intervals that differ by more than 50ms
pNN50	The percentage of NN50
***Frequency domain***	
HF	Power band encompassing 0.15–0.4 Hz range
LF	Power band encompassing 0.04–0.15 Hz range
VLF	Power band encompassing 0.003–0.04 Hz range
LF/HF	The ratio between LF and HF

HF, high frequency; LF, low frequency; VLF, very low frequency.

### Time Domain Measures

Time domain HRV features are calculated with simple mathematical methods to measure the amount of variability present in a specific time period in a continuous ECG signal (19). These parameters are based on the time series of R to R interval (RRI) from the ECG signals. The standard deviation of average normal to normal (NN) intervals (SDNN), the root mean square of successive differences (RMSSD), and the percentage of absolute differences in successive NN values greater than 50ms (pNN50) are widely utilized as time-domain HRV indicators. SDNN is known to reflect both sympathetic and parasympathetic functioning, whereas RMSSD and pNN50 are related to parasympathetic functioning (20–22).

### Frequency Domain Measures

Frequency domain provides an assessment of vagal modulation of the RRI, extracted from the ECG. Frequency domain is mostly commonly acquired by fast Fourier transformation to separate RRI into characteristic very low frequency (VLF, 0.003–0.04 Hz), low frequency (LF, 0.04–0.15 Hz), and high frequency (HF, 0.15–0.4 Hz) band (20). Spectral measures are acquired over different time intervals (approximately 2.5 to 15 min), depending on the frequency being analyzed (20). According to previous studies, LF is influenced by both sympathetic and parasympathetic activities, and HF is affected by mostly parasympathetic activities (23, 24). LF/HF ratio is ratio of LF and HF, and it implicates the sympathetic predominance compared to parasympathetic activities (21, 22).

## HRV for the Diagnosis of MDD

MDD is assoicated with the increased risk of cardiovascular morbidity and mortality (4, 5), and also known to be associated with reduced HRV (25, 26). For these reasons, there have been numerous studies to find the neurobiological biomarkers of MDD related to HRV parameters (**Table 2**).

**Table 2:** Heart rate variability for the diagnosis of major depressive disorder.

Reduced HRV	Kemp et al. (25), Kemp and Quintana, 2013
Increased LF/HF ratio	Udupa et al. (27), Kemp et al. (25), Kemp et al. (26), Choi et al. (28)
Lower HF	Rottenberg (29, 30), Licht et al. (31), Kemp et al. (25)
Lower pNN50	Wang et al. (32), Ha et al. (33), Choi et al. (28)

HF, high frequency; pNN50, the percentage of absolute differences in successive NN values greater than 50 ms.

### Increased LF/HF Ratio

Consistently, there have been many reports which showed an increased LF/HF ratio in patients with MDD compared to HCs (25–28). According to the previous meta-analysis by Kemp et al. which compared 673 depressed patients and 407 healthy controls (HCs) with using 18 articles (25), depressed patients without cardiovascular disease showed reduced time domain HRV, HF HRV, and increased LF/HF ratio than HCs. Udupa et al. also found that 40 patients with MDD showed a more increased LF/HF ratio than 40 age matched HCs (27). More recently, Choi et al. found that patients with MDD showed an elevated LF/HF ratio especially after the stress induction compared HCs (28). The LF/HF ratio is associated with sympathetic predominance (34), which could be related to the increased sympathetic modulation or disrupted ANS modulation in MDD.

### Low HF

As well as LF/HF ratio, decreased HF has also been one of the consistent HRV parameters which were significantly associated with MDD (25, 29, 30, 35, 36). In one meta-analysis, patients with MDD had lower resting levels of HRV than HCs (25). According the large-scale prospective Netherland Study of Depression and Anxiety (NESDA), Licht et al. also showed that remitted and current MDD patients had a lower HF compared to HCs, although they concluded the association appeared to be mainly associated with the effect of antidepressants (36). Rottenberg et al. also found significantly reduced HF in patients with MDD, however the overall effect size was relatively small according to their meta-analysis (29). In reviewing previous reports, HF could be particularly related with anxious depression. Reduction in HF might have significant relations with anxiety according to neurovisceral integration (NVI) model studies (37, 38). Moreover, various anxiety disorders were associated with reduced HRV (39, 40). Some investigators found that low HF in MDD is driven or exacerbated by co-occurring anxiety (26, 31, 41, 42). HF is associated with the parasympathetic tone (43, 44). Relatively high HF is known to reflect adaptive functioning, and neural activity in the prefrontal cortex related to emotional, cognitive, and autonomic regulation (38, 44). Further studies will be needed to evaluate the relationship between HF and MDD.

### Low pNN50

The pNN50 is known to be associated with HF, which reflects the activity level of the parasympathetic nervous system (15, 28). There have been several reports which showed reduced pNN50 in patients with MDD compared to HCs (28, 32, 33). Especially, Ha et al. showed that reduced in pNN50 in the medication-naïve, and newly diagnosed with elderly MDD patients who were older than 60 years old (33). Choi et al. also revealed a lower value of pNN50 compared to HCs at the stress phase, and recovery phase (28).

## Baseline HRV Parameters for the Prediction of Treatment Response in MDD

Antidepressants have been utilized as a front-line treatment of MDD, whereas only one-third to one-half MDD patients who take a complete initial course of antidepressants achieve remission (45, 46). Whereas there are consistent findings that tricyclic antidepressants reduce HRV, it is controversial whether selective serotonin reuptake inhibitors (SSRIs) alters HRV (25, 47). Although there have been studies which found increases in HRV or stability after successful antidepressant treatment in patients with MDD (48, 49), there has been paucity of research which found specific baseline HRV parameters, which could predict treatment responsivity in patients with MDD. **Table 3** summarizes previous HRV findings associated with MDD.

**Table 3:** Heart rate variability for the prediction of better or worse treatment response in MDD.

HRV measures	Related studies
Baseline LF, LF/HF response to the emotional stimuli	Fraguas et al. (50) (Fluoxetine treatment)
Baseline VLF	Jain et al. (51)
Higher HF, and Lower LF	Shapiro et al., (52) (Yoga treatment)
Higher Delta (Stress–Rest phase) LF/HF ratio, pNN50	Choi et al. (28)
Higher baseline HF	Kircanski et al. (31) (Anxious depression)

HF, high frequency; LF, low frequency; VLF, very low frequency; pNN50, the percentage of absolute differences in successive NN values greater than 50 ms.

Previously, in response to the emotional stimulus, baseline changes in LF and LF/HF ratio were positively associated with the decrease level in MDD symptoms during fluoxetine treatment (50). More recently, Jain et al. found that baseline VLF was negatively correlated with symptom improvement in depression (51). Shapiro et al. showed that remitters in MDD had significantly more increased HF, and decreased LF than non-remitters during yoga treatment (52). Choi et al. found that delta LF/HF ratio (Stress phase–Rest phase), and delta pNN50 (Stress phase–Rest phase) were significantly positively associated with treatment response (after 12 weeks) in patients with MDD (28). Regarding types of MDD, Kircanski et al. recently showed that patients with higher HRV, or HF had better treatment outcomes especially in anxious depression (31). However, in non-anxious depression, patients with lower HRV had better outcomes (31). Their study implicates that there might be subtype-specific treatment biomarkers in patients with MDD. A similar study. attempted to differentiate treatment response group from non-response group using EEG and HRV (53). However, the researchers did not predict treatment responsivity in depression using HRV parameters, while they could predict treatment outcome in MDD only using the EEG parameter (53). Despite focusing on PTSD diagnosis other than MDD, Minassian et al. showed that high LF/HF ratio (>6.7) before deployment was significantly associated with post-deployment post-traumatic stress disorder (PTSD) in active-duty marines (54).

## Possible Implication

Disrupted autonomic function may be regarded as a serious pathophysiological candidate for elevated risk of cardiovascular mortality in patients with MDD. Thayer and Lane suggested a neurovisceral integration (NVI) model in the context of emotional regulation (55). According to the NVI model, decreased activation of the central autonomic network (CAN) may affect the decreased level of HRV. CAN is known to be the constellation of brain areas responsible for the neurobiological and physiological regulation of affect and attendant behaviors. According to the NVI modes, the CAN modulates the neuroendocrine, visceromotor, and even behavioral systems (37, 56). Furthermore, the CAN has connection with the sinoatrial node of the heart ***via*** the stellate ganglion through vagus nerve (57). Therefore, HRV is a widely utilized biomarkers of CAN regulatory functioning and considered an informative indicator of brain–body integration, and concomitant health or pathological states (58, 59). CAN is known to consist with the anterior cingulate cortex, insular cortex, ventromedial prefrontal cortex, and the various subcortical structures such as amygdala, hypothalamus, periaqueductal gray matter, parabrachial plexus, and etc. (55–57). Both direct and indirect links between frontal cortex and autonomic motor circuits have been known to be responsible for both the sympathetic and parasympathetic effects on the heart (21, 37, 56). Previous brain imaging studies found that brain regions such as right superior prefrontal, right dorsolateral prefrontal, right dorsolateral prefrontal and left rostral anterior cingulate cortices showed significantly functional decrease concomitantly with decreased HRV (37, 60–62). According to the Thayer and Lane, prefrontal top-down inhibitory and regulatory processes might influence on subcortical emotion regulation centers (37). MDD can be related to the prefrontal hypoactivation and the loss of inhibitory neural functioning with poor affective information processing and regulation (21, 37, 55, 56, 63). Prefrontal hypoactivity might be associated with altered cardiac function in MDD patients, specifically for treatment non-responders.

## Methodologic Consideration

Although HRV is a non-invasive, pain free, economic and simple technique and one of the easily accessible modalities measuring ANS profiles (18), it is important to consider several important potential confounding factors for the future research.

### Time of Measurement

Due to circadian variation in autonomic cardiac function and HRV (64, 65), it should be recommended to evaluate HRV parameters at about the same time of the day. Furthermore, participants should be recommended to have a normal sleep routine, no intense physical training, and no alcohol the day before the measurement (66–70).

### Demographic Factors: Age, Gender, Alcohol Use, Smoking and Body Weight

According to the previous research, HRV decreases with aging (71), or HRV parameters changes with a trend toward a decrease in autonomous cardiac function (72, 73). HRV parameters are also known to have different profiles between male and female population (71, 72, 74). In the recent meta-analysis, Koenig and Thayer showed that females had a significantly lower mean RR interval, lower SDNN, lower LF power, lower LF/HF ratio and greater HF power, which implied more increased parasympathetic activity than males (74). Alcohol use is also associated with altered HRV parameters (69, 70). According to the meta-analytic study by Quintana et al., alcohol dependence patients showed reduced HRV compared to nondependent controls (69). On the contrary, the researchers found that habitual, and moderate alcohol drinkers showed increased levels of HF compared to nonhabitual drinkers in their other original study (70). It might be associated with a J-shaped curve that moderate alcohol use is related to a protective effect compared to alcohol dependence or abstinence (69, 70). Smoking is also associated with reduced HRV levels according to previous studies (75, 76). Recent studies also reported that even e-cigarette use decreased HF component, and increased LF and LF/HF ratio compared to controls (77, 78). Weight, height, and waist-to-hip ratio are also considered as potential confounding factors (79). Yi et al. recently showed that waist-to-hip ratio was more strongly correlated with HRV indices and more likely predict reduced HRV compared to body mass index (BMI), and percentage of body fat mass. However, although the previous study indicated no correlation between HRV and BMI (80), BMI should be considered as one of the confounding factors since it is still controversial (79, 81). Therefore, above-mentioned demographic factors should be considered to conduct future research related to HRV.

### Antidepressant Medication

Previous studies suggest HRV alterations related to antidepressant medication. According to 2010 Kemp et al.**’**s meta-analysis, they showed that tricyclic antidepressant (TCA) decreased HRV whereas SSRI nefazedone, and mirtazapine did not have any significant effect on HRV (25). On the contrary, their large-scale longitudinal study showed that SSRI, and serotonin and norepinephrine reuptake inhibitors decreased HRV parameters (82). More recently, without TCA and clozapine, there were no significant effect on HRV parameters associated with SSRI treatment (83). Futures studies will be needed to clarify relationships between specific treatment regimen and HRV parameters.

## Conclusion

In conclusion, there have been several attempts to diagnose MDD, and to predict treatment responsiveness in patients with MDD with using baseline HRV parameters. We should consider methodological issues and potential confounding factors to examine relationships between MDD and HRV parameters. Furthermore, it will be needed to have larger sample size, prospective and longitudinal study design, and related other regimen such as neuroimaging, inflammatory markers, and so on for the more refined future research.

## Author Contributions

KC and HJ: Writing and reviewing the whole manuscript.

## Conflict of Interest

The authors declare that the research was conducted in the absence of any commercial or financial relationships that could be construed as a potential conflict of interest.
